# Synaptic activity is not required for establishing heterogeneity of inner hair cell ribbon synapses

**DOI:** 10.3389/fnmol.2023.1248941

**Published:** 2023-09-06

**Authors:** Nare Karagulyan, Tobias Moser

**Affiliations:** ^1^Institute for Auditory Neuroscience and InnerEarLab, University Medical Center Göttingen, Göttingen, Germany; ^2^Auditory Neuroscience and Nanophysiology Group, Max Planck Institute of Multidisciplinary Sciences, Göttingen, Germany; ^3^Collaborative Research Center 889, University of Göttingen, Göttingen, Germany; ^4^Göttingen Graduate School for Neurosciences and Molecular Biosciences, University of Göttingen, Göttingen, Germany; ^5^Hertha Sponer College, Multiscale Bioimaging Cluster of Excellence (MBExC), University of Göttingen, Göttingen, Germany; ^6^Multiscale Bioimaging Cluster of Excellence (MBExC), University of Göttingen, Göttingen, Germany

**Keywords:** hearing, cochlea, ribbon synapse, Ca^2+^ channels, spiral ganglion neuron, sound encoding

## Abstract

Neural sound encoding in the mammalian cochlea faces the challenge of representing audible sound pressures that vary over six orders of magnitude. The cochlea meets this demand through the use of active micromechanics as well as the diversity and adaptation of afferent neurons and their synapses. Mechanisms underlying neural diversity likely include heterogeneous presynaptic input from inner hair cells (IHCs) to spiral ganglion neurons (SGNs) as well as differences in the molecular profile of SGNs and in their efferent control. Here, we tested whether glutamate release from IHCs, previously found to be critical for maintaining different molecular SGN profiles, is required for establishing heterogeneity of active zones (AZs) in IHCs. We analyzed structural and functional heterogeneity of IHC AZs in mouse mutants with disrupted glutamate release from IHCs due to lack of a vesicular glutamate transporter (Vglut3) or impaired exocytosis due to defective otoferlin. We found the variance of the voltage-dependence of presynaptic Ca^2+^ influx to be reduced in exocytosis-deficient IHCs of otoferlin mutants. Yet, the spatial gradients of maximal amplitude and voltage-dependence of Ca^2+^ influx along the pillar-modiolar IHC axis were maintained in both mutants. Further immunohistochemical analysis showed an intact spatial gradient of ribbon size in Vglut3^–/–^ mice. These results indicate that IHC exocytosis and glutamate release are not strictly required for establishing the heterogeneity of IHC AZs.

## 1. Introduction

The mammalian auditory system responds to sound pressures ranging over six orders of magnitude. Downstream of dynamic range compression by active cochlear micromechanics, inner hair cells (IHCs)—the sensory receptors—represent the entire audible range. Yet, the rate of sound-evoked firing in each of their postsynaptic spiral ganglion neurons (SGNs) encodes only a fraction of it. SGNs tuned to a given sound frequency differ in their molecular profile and morphology as well as spontaneous and sound-evoked firing. They tile the audible intensity range and segregate their central projections. SGNs are often classified according to their spontaneous rate (SR) into high, medium, and low SR fibers (Kiang et al., 1965; Sachs and Abbas, 1974; Liberman, 1978; Winter et al., 1990). High SR fibers display low sound thresholds and therefore encode soft sounds, while low SR fibers with higher thresholds present stronger sound intensities. Recent transcriptomic studies identified three molecularly distinct classes of type I SGNs (I_a_, I_b_, I_c_), which have been suggested to correspond to the above described physiological subtypes of SGNs (Petitpré et al., 2018; Shrestha et al., 2018; Sun et al., 2018; Li et al., 2020). Differences in type I SGNs are also reflected in the spatial innervation pattern of their presynaptic partner IHCs. Each IHC is innervated by 5–30 type I SGN (review in Meyer and Moser, 2010), whereby low SR and high threshold SGNs are said to predominantly contact the modiolar (neural) side of the IHC as opposed to the high-spontaneous rate low threshold SGNs, which primarily synapse on the pillar (abneural) side of the cell (Liberman, 1982; Merchan-Perez and Liberman, 1996). Similarly, SGNs with type I_a_ molecular profile preferentially synapse on the pillar side of an IHC (Shrestha et al., 2023; Siebald et al., 2023), while type I_b/c_ SGNs synapse on the modiolar side (Sherrill et al., 2019) and have lower SR (Siebald et al., 2023).

Interestingly, the AZs within an IHC display highly variable size, amplitude, and voltage dependence in terms of Ca^2+^ influx, glutamate release, Ca^2+^ influx-release coupling (Frank et al., 2009; Meyer et al., 2009; Liberman et al., 2011; Neef et al., 2018; Hua et al., 2021; Özçete and Moser, 2021). Those properties exhibit gradients along the modiolar-pillar axis of the IHCs, with modiolar AZs showing larger ribbons and higher maximal amplitude yet more depolarized Ca^2+^ influx and consequently glutamate release, requiring stronger depolarization (Ohn et al., 2016; Özçete and Moser, 2021). Therefore, it has been hypothesized that a single IHC fractionates the entire sound input into corresponding neural codes through heterogeneous presynaptic active zones (AZs, Frank et al., 2009; Ohn et al., 2016; Özçete and Moser, 2021).

The molecular mechanisms that shape the heterogeneity of AZs in IHCs remain largely unknown. Previous studies provided preliminary evidence for an impact of efferent innervation (Yin et al., 2014; Hickman et al., 2015), transsynaptic signaling (Sherrill et al., 2019; Shrestha et al., 2023), and intrinsic planar polarity signaling (Jean et al., 2019). Moreover, disruption of the key AZ protein bassoon reduced the extent of AZ heterogeneity (Frank et al., 2010). Other manipulations, such as disruption of the AZ proteins RIM, RIM-BP, and RIBEYE or impaired mechanotransduction, affected synapses globally yet without obviously altering AZ heterogeneity (Jung et al., 2015; Krinner et al., 2017, 2021; Becker et al., 2018; Jean et al., 2018). Yet, to our knowledge, the impact of abolished IHC exocytosis has not been tested.

Here, we explored the hypothesis that synaptic transmission is relevant for establishing AZ heterogeneity and setting the spatial gradients of AZ properties along the modiolar-pillar axis of the IHCs. For that purpose, we used mice lacking the vesicular glutamate transporter 3 (Vglut3) as a model for abolished glutamatergic signaling from IHCs despite maintained exocytosis (Ruel et al., 2008; Seal et al., 2008). Previous studies on Vglut3 KO mice have shown that glutamatergic transmission at the afferent IHC synapse is required for maintaining molecular identities of type I_*b*_ and I_*c*_ SGNs (Shrestha et al., 2018; Sun et al., 2018). The changes in subtype specification might lead to changes in presynaptic properties via transsynaptic signaling, as shown for postnatal disruption of the transcription factor *Pou4f1* that is specific for type I_*c*_ and I_*b*_ SGNs (Sherrill et al., 2019). In addition, we employed a new mouse mutant with disrupted IHC exocytosis due to mutations in the *Otof* gene (*Otof^TDA/TDA^*). *Otof^TDA/TDA^* mice harbor three *Otof* mutations that disrupt Ca^2+^ binding to the C_2_E domain of otoferlin and show abolished Ca^2+^ influx-triggered exocytosis despite near normal abundance and distribution of otoferlin as well as maintained synapses at the age of 3 weeks.

Analyzing ribbon size and Ca^2+^ influx at individual presynaptic AZs of *Vglut3*^–/–^ and *Otof^TDA/TDA^* IHCs, we found the spatial gradients for the amplitude and voltage dependence of Ca^2+^ influx as well as the gradient of the ribbon size to be preserved. Despite the intact gradients, the variability of the voltage of half maximal activation among the AZs of *Otof^TDA/TDA^* IHCs was significantly lower compared to that of the WT IHCs.

## 2. Materials and methods

### 2.1. Animals

All the experiments described in the study were performed in P21–P28 mice of either sex. *Vglut3*^–/–^ mice have been described before (Ruel et al., 2008) and the generation of *Otof^TDA/TDA^* will be described in a manuscript (Chen et al.) that is currently under peer review elsewhere. In brief, a CRISPR/Cas9 approach was employed and ribonucleoprotein particles were injected into C57Bl6N oocytes. Correct editing of *Otof* was validated in founder (F0) mice, which were then crossed with C57Bl6N mice. Germline transmission was confirmed by heterozygosity for the edited allele. F2 mice were born at a Mendelian ratio, and heterozygous breeding was used to generate *Otof^TDA/TDA^* and WT littermates for experiments. *Otof^TDA/TDA^* were found to be deaf by recordings of auditory brainstem responses but seemed otherwise fine according to routine observation. Breeding was done in compliance with the German national animal care guidelines and approved by the local animal welfare committee of the University Medical Center Göttingen and the Max Planck Institute for Multidisciplinary Sciences, as well as the animal welfare office of the state of Lower Saxony, Germany (LAVES, AZ 19/3134).

### 2.2. Patch clamp and Ca^2+^ imaging

We dissected the apical coils of the organs of Corti, and patch-clamped IHCs at the tonotopic location of around 6–8 kHz from either pillar or modiolar side. Patch pipettes (Science products, GB150F-8P) were pulled with a P-97 Flaming/Brown micropipette puller (Sutter Instruments). Intracellular solution contained (in mM): 111 Cs-glutamate, 1 MgCl_2_, 1 CaCl_2_, 10 EGTA, 13 TEA-Cl, 20 HEPES, 4 Mg-ATP, 0.3 Na-GTP, 1 L-glutathione, 0.8 Fluo-4FF (Life Technologies), and 0.01 TAMRA-conjugated CtBP2/RIBEYE binding peptide, pH 7.3, 290 mOsm. The extracellular solution contained the following (in mM): 2.8 KCl, 105 NaCl, 10 HEPES, 1 CsCl_2_, 1 MgCl_2_, 5 CaCl_2_, 35 TEA-Cl, and 2 mg/ml D-glucose, pH 7.2, 300 mOsm. Recordings were performed at room temperature (22–23°C).

Data were acquired with an EPC-10 amplifier controlled by PatchMaster software (HEKA Elektronik). The holding potential of the cell was set to −87 mV. Whole cell Ca^2+^ influx was triggered by applying either voltage step or voltage ramp depolarizations to the cell. Voltage step depolarizations were applied from −82 to 63 mV with 5 mV increments and voltage ramp depolarizations ranged from −87 to 63 mV over the course of 150 ms (1 mV/ms). Recordings were leak corrected using P/n protocol. All voltages were corrected offline for voltage drops across series resistance (R_*s*_) and liquid junction potential, which was calculated to be 17 mV. Recordings were discarded from analysis in case they displayed leak currents beyond −50 pA at the holding potential (−87 mV), R_*s*_ above 14 MOhm during the first 3 min after rupturing the cell, or Ca^2+^ current rundown above 25% by the end of the experiment.

Ca^2+^ imaging from IHCs was described previously (Ohn et al., 2016; Cantu-Guerra et al., 2023). Briefly, a spinning disk confocal scanner (CSU22, Yokogawa) mounted on an upright microscope (Axio Examiner, Zeiss) and a 1.0 NA 63x objective (W Plan-Apochromat, Zeiss) were used. Image acquisition was performed with a sCMOS camera (Neo, Andor). The spinning disk speed was set to 2000 rpm.

Z-stacks during the live imaging were acquired using a fast piezoelectric system (Piezosystem). To obtain the morphology of the IHC and identify the positions of the AZs, we first acquired a Z-stack of the cell through imaging TAMRA fluorescence using a 591 nm laser (Cobolt AB). Each plane was exposed for 0.5 s and the step size for Z-scanning was 0.5 μm. Ca^2+^ imaging was performed by exciting Fluo-4FF with 491 nm laser (Cobolt AB). Ca^2+^ imaging was restricted to the planes which contained ribbons. Hotspots of Fluo-4FF fluorescence increases were evoked by voltage ramp depolarizations and simultaneously imaged at a frame rate of 100 Hz at each plane. For each plane, two different voltage ramps were applied, one being 5 ms shifted relative to the other one. This served the purpose of increasing the voltage resolution of Ca^2+^ imaging when later concatenating the two fluorescence traces evoked by the two different voltage ramps.

### 2.3. Immunohistochemistry and imaging

The samples were fixed in 4% formaldehyde on ice. Time of fixation was 10 min whenever anti-Ca_*v*_1.3 antibody was used; for the rest of the stainings, the samples were fixed for 60 min. The following primary antibodies were used: mouse anti-Ctbp2 (1:200, BD Biosciences, 612044), rabbit anti-Cav1.3 (1:100, Almone Labs, ACC005), and rabbit anti-Myosin 7a (1:200, Enzo Life Sciences, PTS-25-6790-C050). Afterward, the following secondary antibodies were added: Alexa Fluor 488 conjugated anti-rabbit (1:200, Invitrogen, A11008) and Alexa Fluor 633 conjugated anti-mouse (1:500, Invitrogen, A21126). For superresolution STED imaging STAR580 conjugated anti-mouse (1:200, Abberior, 2-0002-005-1) and STAR635 (1:200, Abberior, 2-0012-007-2) conjugated anti-rabbit secondary antibodies were used. In order to prevent the hair cells from flattening due to the weight of the coverslip, 50 μm plastic films were taped between the coverslip and the microscopy slide from both sides of the sample. Imaging was performed in confocal or 2D STED mode using the Abberior Instruments Expert line STED microscope equipped with 488 nm, 561 nm, and 633 nm lasers and a 775 nm STED laser. A 1.4 NA 100x oil immersion objective was used.

### 2.4. Data analysis

#### 2.4.1. Patch-clamp and imaging

Data were analyzed with Igor pro 6.3 software (Wavemetrics) using custom written procedures. Whole-cell current–voltage relationships (IV curves) were analyzed by plotting the evoked Ca^2+^ currents averaged over the 5–10 ms interval after the start of stimulation against the depolarization voltages.

The analysis of Ca^2+^ imaging was performed as described previously (Cantu-Guerra et al., 2023). Briefly, Ca^2+^ hotspots were visualized by subtracting the average of 10 resting frames from the average of 5 frames during stimulation. To obtain the intensity profile of Fluo4-FF fluorescence over time the intensity of the central pixel of the hotspot was averaged with the 8 surrounding pixels at all-time points. Two intensity profiles corresponding to the shifted voltage ramp stimuli were concatenated and plotted against voltage (FV curve). The FV curves were fitted using modified Boltzmann function:


$$F=F_{0}+\frac{G_{m\,a\,x}\,\left(V_{r\,e\,v}-V_{m}\right)}{1+\exp\left[\frac{V_{m}-V_{h}}{k}\right]}$$

where F is the fluorescence intensity at a given voltage, F_0_ is the fluorescence at the resting state, V_*rev*_ is the reversal potential calculated from the IV recordings of the whole cell patch-clamp data, V_*m*_ is the cell membrane voltage, V_*h*_ is the voltage of half-maximal Ca^2+^ influx, and k is the slope.

Fractional activation curves of the Ca^2+^ channels at single active zones were calculated by dividing FV curves by the lines fitted to the fluorescence decay of the FV curves at depolarized voltages (G_max_). Resulted curves were fitted with the Boltzmann function (Figure 3A).

To analyze the position dependency of Ca^2+^ influx properties at individual IHC AZs, the coordinates of the ribbons (visualized by TAMRA-conjugated dimeric Ctbp2 binding peptide) were transferred from the canonical Cartesian coordinate system into a self-defined polar coordinate system (Ohn et al., 2016; Cantu-Guerra et al., 2023). Briefly, we performed 3D reconstruction of the cells, whereby the symmetry vector of the cell (V_*sym*_, orthogonal vector to the plane of symmetry) as well as the central vector of the cell (V_*z*_, connecting the centers of mass of the bottom-most plane and the largest plane) were calculated. Afterward we obtained the vector defining modiolar-pillar axis (V_*pm*_) of the cell by multiplying V_*sym*_ and V_*z*_. Finally, the polar coordinates of the ribbons were calculated and plotted in polar charts. The two orthogonal axes of the polar charts represent apical-basal (referring to the tonotopic axis) and pillar-modiolar axes of the cell.

#### 2.4.2. Analysis of confocal and STED imaging data

For the position-dependent analysis of ribbon size, we used a customized MATLAB plugin for the Imaris software, as it was described before (Jean et al., 2019; Sherrill et al., 2019). Briefly, Ctbp2 immunofluorescent spots were automatically detected on Imaris, and the quality of detection was adjusted subjectively. The intensities of the ribbons were calculated as the sum of pixel intensities of the 7x7x5 region around the center of mass of the immunofluorescent spots. Based on the cytosolic and nuclear staining, the central vector of the cell was assigned by connecting the base of the cell to the center of the nucleus of the cell and was further adjusted XY and YZ planes. The Cartesian coordinates of the ribbons, which were defined as the coordinates of the center of the masses of the 3D Gaussian fits were transformed to the polar coordinate system. Ribbon immunofluorescence intensities of each sample were normalized to the median fluorescence of the modiolar ribbons.

2D STED images of the ribbons and Ca_*v*_1.3 line clusters were analyzed with Igor Pro software and were fitted with 2D Gaussian function to obtain full with at half maxima (FWHM) of long and short axes using genetic fit algorithm (Sanchez del Rio and Pareschi, 2001). Confocal and STED images were adjusted for visualization purposes using Fiji software.

#### 2.4.3. Statistical analysis

Data were analyzed using Igor Pro software. Data is presented as mean ± standard error of the mean (SEM), and the standard deviation (SD) is shown for each data set. The number of the animals is indicated as N. For two sample comparison, normality of the distributions (Jarque-Bera test) and equality of variances (*F*-test) were tested. This was followed by Student’s *t*-test or Mann-Whitney-Wilcoxon test in case the normality of distributions and/or equality of variances were not met. Levene’s test was used to test the equality of variances between the distributions of voltage of half maximal activations of Ca^2 +^ influx in WT and *Otof*^*TDA*/*TDA*^ IHCs. Significant differences are presented as *p < 0.05, **p < 0.01, ***p < 0.001.

## 3. Results

### 3.1. Heterogeneity of Ca^2+^ influx at the AZs of *Vglut3*^–/–^ IHCs

**FIGURE 1:**
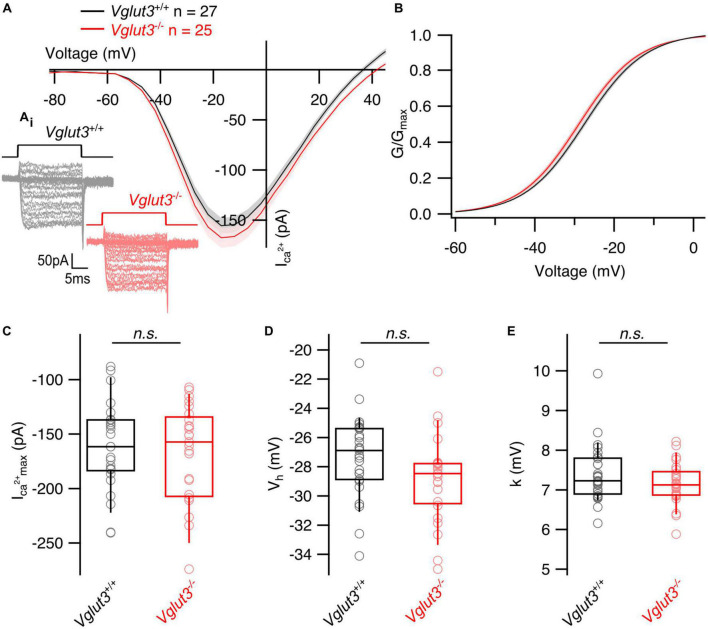
Whole cell Ca^2+^ currents are not changed in inner hair cells of *Vglut3*^–/–^ mice. **(A)** Average whole cell current–voltage relationships obtained by recording Ca^2+^ influx in response to step depolarizations from the IHCs of *Vglut3^+/+^* and *Vglut3*^–/–^ animals. Shaded areas show ± SEM. **(A_i_)** Representative Ca^2+^ current traces from *Vglut3*^+/+^ and *Vglut3*^–/–^ IHCs evoked by step depolarizations ranging from –82 to 63 mV with 5 mV increment (top). **(B)** Average fractional activation curves calculated from IV relationships of *Vglut3^+/+^* and *Vglut3*^–/–^ IHCs and fitted with Boltzmann function. Shaded areas show ± SEM. **(C)** Box plots show no difference in the peak Ca^2+^ current recorded from IHCs of *Vglut3^+/+^* and *Vglut3*^–/–^ mice (*Vglut3^+/+^*: –161 ± 7.87 pA, SD = 40.12 pA, *n* = 27, *N* = 16; *Vglut3*^–/–^: –170 ± 9.79 pA, SD = 48.96 pA, *n* = 25, *N* = 12; Student’s *t*-test, *p* = 0.49). **(D)** Box plots show no difference in the voltage of half maximal activation obtained from fractional activation curves of *Vglut3^+/+^* and *Vglut3*^–/–^ IHCs (*Vglut3^+/+^*: –27.3 ± 0.53 mV, SD = 2.75 mV, *n* = 27, *N* = 16; *Vglut3*^–/–^: –28.8 ± 0.59, SD = 2.95 mV, *n* = 25, *N* = 12; Student’s *t*-test, *p* = 0.06). **(E)** Voltage sensitivity of Ca^2+^ influx shows no difference between IHCs of *Vglut3^+/+^* and *Vglut3*^–/–^ mice (*Vglut3^+/+^*: 7.39 ± 0.14 mV, SD = 0.73 mV, *n* = 27, *N* = 16; *Vglut3*^–/–^: 7.16 ± 0.11, SD = 0.53 mV, *n* = 25, *N* = 12; Mann-Whitney-Wilcoxon test, *p* = 0.31). Box-whisker plots with individual data points overlaid show median (middle line), 25^th^ and 75^th^ percentiles (lower and upper borders of the box) and 10^th^ and 90^th^ percentiles (whiskers).

Transcriptomic studies of single SGNs in *Vglut3*^–/–^ mice revealed an altered molecular subtype specification of SGNs: 80% of the neurons gained type I_*a*_ identity, while the proportion of type I_*b*_ and I_*c*_ neurons was down to 20% (Shrestha et al., 2018; Sun et al., 2018). This has been taken to indicate that postnatal glutamatergic transmission at the afferent IHC synapse is required to maintain the molecular identity of type I_*b*_ and I_*c*_ SGNs. Here, we asked the question if changes in synaptic transmission and/or altered transsynaptic signaling from SGNs affect the presynaptic heterogeneity of IHCs. To do so, we assessed the functional heterogeneity of IHC AZs by combining IHC patch-clamp and Ca^2+^ imaging at IHC AZs in *Vglut3*^–/–^ mice at 3 weeks of age. Whole cell Ca^2+^ currents tended to be slightly larger in *Vglut3*-deficient IHCs without reaching significance (*Vglut3^+/+^*: −161 ± 7.87 pA, SD = 40.12 pA, *n* = 27, *N* = 16 vs. *Vglut3*^–/–^: −170 ± 9.79 pA, SD = 48.96 pA, *n* = 25, *N* = 12; Student’s *t*-test, *p* = 0.49, Figures 1A, C), while a previous study showed a significantly increased Ca^2+^ influx in Vglut3-deficient IHCs (Ruel et al., 2008). A potential explanation for this discrepancy is the age difference of mice. While an increase in the Ca^2+^ current amplitude was previously reported for p12–p18 mice, we used 3-week-old mice in our study, at which stage a reduced synapse number might mask the increased Ca^2+^ current. The voltage dependence of Ca^2+^ channel activation and its voltage sensitivity, approximated as the slope of the Boltzmann function fit to the fractional activation curve, were not significantly altered, but there was a trend toward activation at lower potentials for *Vglut3*-deficient IHCs (Figures 1B, D, E).

**FIGURE 2:**
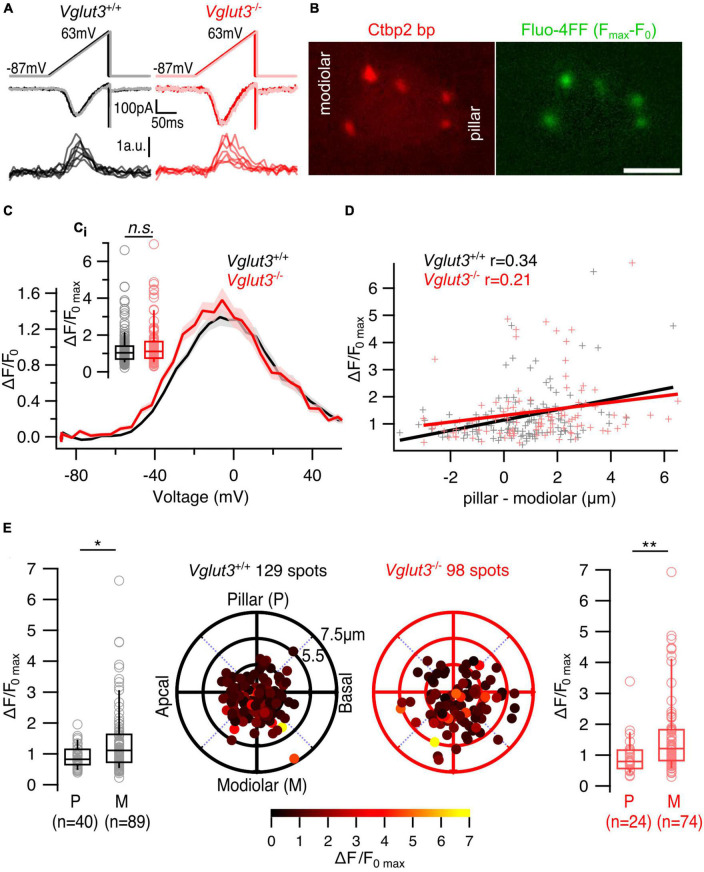
The spatial gradient of maximum Ca^2+^ influx is preserved in IHCs of *Vglut3*^–/–^ mice. **(A)** Representative whole cell Ca^2+^ currents and single synaptic Ca^2+^ influx fluorescence traces evoked by voltage ramp depolarizations. **(B)** Single plane of an IHC filled with TAMRA-conjugated Ctbp2 binding peptide and Fluo-4FF shows synaptic ribbons (left) and corresponding Ca^2+^ hotspots (right). Scale bar = 5 μm **(C)** Average ΔF/F_0_ traces of single AZs plotted against depolarization voltages (FV curves). Shaded areas represent ± SEM. **(C_i_)** Box plots show no difference in single AZ maximal Ca^2+^ influx (ΔF/F_0max_) of *Vglut3^+/+^* and *Vglut3*^–/–^ IHCs (*Vglut3^+/+^*: 1.25 ± 0.083, SD = 0.94, 129 spots, *n* = 27, *N* = 16; *Vglut3*^–/–^: 1.45 ± 0.12, SD = 1.18, 98 spots, *n* = 20, *N* = 12; Mann-Whitney-Wilcoxon test, *p* = 0.22). **(D)** Single AZ maximal Ca^2+^ influx plotted against the position of the AZ along the modiolar-pillar axis of the cell. AZ position was calculated by projecting its polar coordinates onto the pillar-modiolar axis. Zero μm shows the center of the imaged IHC plane, in which the synaptic ribbon was detected. Solid lines represent the linear regression curves and r shows the Pearson’s correlation coefficient. **(E)** Polar plots show the locations of individual AZs in *Vglut3^+/+^* (left, black) and *Vglut3*^–/–^ (right, red) IHCs. Pseudo-color scale shows ΔF/F_0max_. Box plots compare ΔF/F_0max_ of pillar and modiolar AZs and show larger Ca^2+^ influx at modiolar AZs compared to the pillar AZs in both *Vglut3^+/+^* (pillar: 0.91 ± 0.056, SD = 0.36, 40 spots; modiolar: 1.4 ± 0.11, SD = 1.08, 89 spots; Mann-Whitney-Wilcoxon test, *p* = 0.01) and *Vglut3*^–/–^ (pillar: 0.98 ± 0.13, SD = 0.64, 24 spots; modiolar: 1.61 ± 0.16, SD = 1.28, 74 spots; Mann-Whitney-Wilcoxon test, *p* = 0.004) IHCs. Box-whisker plots with individual data points overlaid show median (middle line), 25^th^ and 75^th^ percentiles (lower and upper borders of the box) and 10^th^ and 90^th^ percentiles (whiskers). Asterisks indicate statistical significance (*p < 0.05, **p < 0.01).

Next, we imaged synaptic Ca^2+^ signals at single IHC AZs using spinning disk confocal microscopy to analyze the heterogeneity of Ca^2+^ influx among the AZs (Ohn et al., 2016; Jean et al., 2019; Sherrill et al., 2019). IHCs were loaded with a TAMRA-conjugated Ctbp2-binding dimeric peptide that fluorescently labels synaptic ribbons (Zenisek et al., 2004) and the Ca^2+^ indicator Fluo-4FF (K_*D*_ ∼10 μM). Scanning the IHC from the basal end to the cuticular plate using a 561 nm laser obtained the IHC morphology and detected the ribbon containing confocal planes, at which we next imaged (491 nm laser) the synaptic Ca^2+^ signals upon voltage ramp depolarization. Employing strong intracellular Ca^2+^ buffering (10 mM EGTA), we interpret the Fluo-4FF fluorescence increase (ΔF/F_0_) to approximate the Ca^2+^ influx at the single AZ (Frank et al., 2009). The maximal Ca^2+^ influx at single AZs (ΔF/F_0*max*_) of *Vglut3*^–/–^ mice tended to be slightly higher compared in IHCs of *Vglut3^+/+^* mice without reaching statistical significance (Figures 2A, B; *Vglut3^+/+^*: 1.25 ± 0.083, SD = 0.94, 129 spots, *n* = 27, *N* = 16; *Vglut3*^–/–^: 1.45 ± 0.12, SD = 1.18, 98 spots, *n* = 20, *N* = 12; Mann-Whitney-Wilcoxon test, *p* = 0.22; Figures 2C, C_i_).

Using 3D reconstruction of the cells we assigned the AZ properties to their positions (see section “2. Materials and methods”). We found mild but significant modiolar-pillar gradient of maximal amplitude of synaptic Ca^2+^ influx (i.e., stronger AZs on the modiolar side of the cell in terms of Ca^2 +^ influx amplitude) in both WT and *Vglut3*^–/–^ IHCs (Figure 2D, Supplementary Table 1; *Vglut3^+/+^*: Pearson’s correlation coefficient *r* = 0.34; *Vglut3*^–/–^: *r* = 0.21). Binary separation into pillar and modiolar AZs showed a significant difference in the maximum Ca^2+^ influx between pillar and modiolar AZs in *Vglut3*^–/–^ IHCs similar to the controls (*Vglut3^+/+^*: pillar: 0.91 ± 0.056, SD = 0.36, 40 AZs; modiolar: 1.4 ± 0.11, SD = 1.08, 89 AZs; Mann-Whitney-Wilcoxon test, *p* = 0.01; *Vglut3*^–/–^: pillar: 0.98 ± 0.13, SD = 0.64, 24 AZs; modiolar: 1.61 ± 0.16, SD = 1.28, 74 AZs; Mann-Whitney-Wilcoxon test, *p* = 0.004; Figure 2E).

To test whether the difference in ΔF/F_0*max*_ between pillar and modiolar AZs in *Vglut3*^–/–^ IHCs was dominated by modiolar AZs with high fluorescence (“winner” AZ, Ohn et al., 2016), we analyzed modiolar-pillar gradient of maximum Ca^2+^ influx without the “winners”. As done before (Ohn et al., 2016), we defined “winner” AZs to have ΔF/F_0*max*_ values at least 2.5 higher than the average ΔF/F_0*max*_ of the rest (Supplementary Figures 1A, B). Interestingly, the modiolar-pillar gradient of maximum Ca^2+^ influx in *Vglut3*^–/–^ IHCs remained without the “winner” spots (Supplementary Figures 1C, D).

**FIGURE 3:**
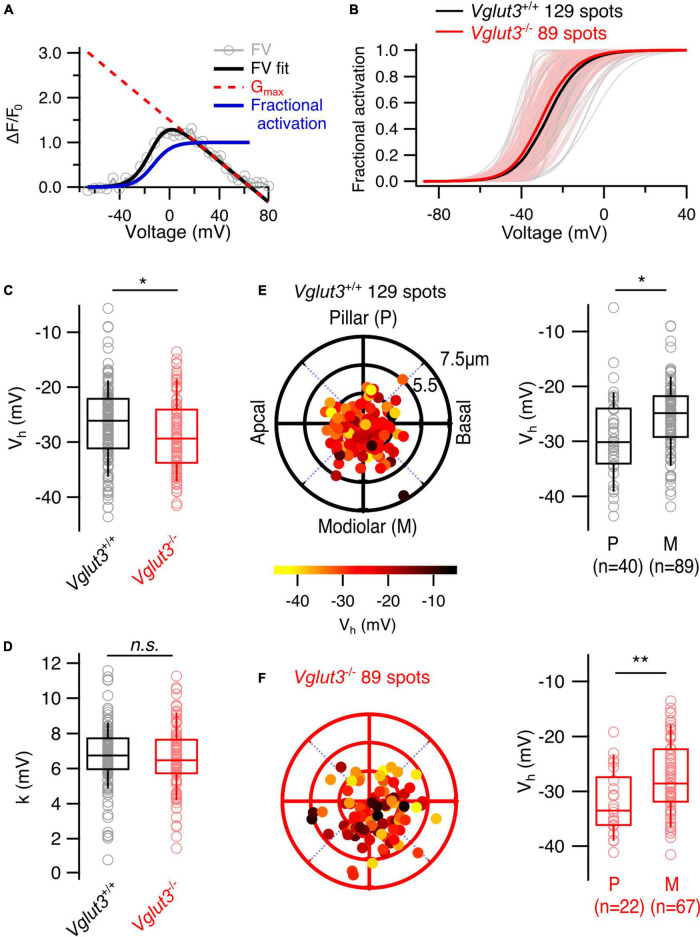
Lower voltage of Ca^2+^ channel activation but preserved pillar-modiolar gradient of voltage of half maximal activation in IHCs of *Vglut3*^–/–^ mice. **(A)** Fractional activation curves of individual fluorescence traces (blue solid line) were calculated by fitting the FV curves with modified Boltzmann function (FV fit, black solid line) and dividing the resulting fits by G_max_ lines (red dotted line), which were obtained by fitting a line to the FV fits in the range of 3 mV to the reversal potential. **(B)** Fractional activation curves of single AZs in *Vglut3^+/+^* and *Vglut3*^–/–^ IHCs. Thick solid lines show the averages. **(C)** Box plots comparing voltage of half maximal activation (V_h_) at single AZ level show mild hyperpolarized shift in IHCs of *Vglut3*^–/–^ mice (*Vglut3^+/+^*: –26.7 ± 0.61 mV, SD = 6.96 mV, 129 spots, *n* = 27, *N* = 16; *Vglut3*^–/–^: –28.7 ± 0.71 mV, SD = 6.7 mV, 89 spots, *n* = 20, *N* = 12; Student’s *t*-test, *p* = 0.03). **(D)** Voltage sensitivity of Ca^2+^ influx at single AZ level shows no difference between IHCs of *Vglut3^+/+^* and *Vglut3*^–/–^ mice (*Vglut3^+/+^*: 6.77 ± 0.15 mV, SD = 1.74 mV, 129 spots, *n* = 27, *N* = 16; *Vglut3*^–/–^: 6.56 ± 0.2 mV, SD = 1.92 mV, 89 spots, *n* = 25, *N* = 12; Mann-Whitney-Wilcoxon test, *p* = 0.26). Polar plots show the locations of individual AZs in *Vglut3^+/+^* **(E)** and *Vglut3*^–/–^ **(F)** IHCs. Pseudo-color scale shows voltage of half maximal activation. Box plots compare V_h_ of pillar and modiolar AZs and show depolarized Ca^2+^ influx at modiolar AZs compared to the pillar AZs in both *Vglut3^+/+^* (pillar: –29.1 ± 1.2 mV, SD = 7.56 mV, 40 spots; modiolar: –25.6 ± 0.11 mV, SD = 0.68 mV, 89 spots; Student’s *t*-test, *p* = 0.01) and *Vglut3*^–/–^ (pillar: –32.1 ± 1.26 mV, SD = 5.9 mV, 22 spots; modiolar: –27.6 ± 0.81 mV, SD = 6.6 mV, 67 spots; Mann-Whitney-Wilcoxon test, *p* = 0.005) IHCs. Box-whisker plots with individual data points overlaid show median (middle line), 25^th^ and 75^th^ percentiles (lower and upper borders of the box) and 10^th^ and 90^th^ percentiles (whiskers). Asterisks indicate statistical significance (*p < 0.05, **p < 0.01).

Next, we analyzed the voltage dependence of Ca^2+^ channel activation at individual AZs by calculating the fractional activation curves from Ca^2+^ fluorescence–voltage relationships (see section “2. Materials and methods,” Figure 3A). We observed a significant hyperpolarized shift of the voltage of half-maximal Ca^2+^ channel activation (V_*h*_) at the single AZ level in IHCs of *Vglut3*^–/–^ mice (*Vglut3^+/+^*: −26.7 ± 0.61 mV, SD = 6.96 mV, 129 AZs, *n* = 27, *N* = 16; *Vglut3*^–/–^: −28.7 ± 0.71 mV, SD = 6.7 mV, 89 AZs, *n* = 20, *N* = 12; Student’s *t*-test, *p* = 0.03; Figures 3B, C) but no change in voltage sensitivity of Ca^2 +^ influx (*Vglut3*^+/+^: 6.77 ± 0.15 mV, SD = 1.74 mV, 129 spots, *n* = 27, *N* = 16; *Vglut3*^–/–^: 6.56 ± 0.2 mV, SD = 1.92 mV, 89 spots, *n* = 25, *N* = 12; Mann-Whitney-Wilcoxon test, *p* = 0.26, Figure 3D). Interestingly, a similar hyperpolarized shift was previously observed in *Pou4F1* cKO mice (Sherrill et al., 2019). Despite the average hyperpolarized shift, the pillar AZs of *Vglut3*^–/–^ IHCs showed a significantly lower V_*h*_ than the modiolar AZs, (*Vglut3^+/+^*: pillar: −29.1 ± 1.2 mV, SD = 7.56 mV, 40 AZs; modiolar: −25.6 ± 0.11 mV, SD = 0.68 mV, 89 AZs; Student’s *t*-test, *p* = 0.01; *Vglut3*^–/–^: pillar: −32.1 ± 1.26 mV, SD = 5.9 mV, 22 AZs; modiolar: −27.6 ± 0.81 mV, SD = 6.6 mV, 67 AZs; Mann-Whitney-Wilcoxon test, *p* = 0.005, Figures 3E, F). Such a pillar-modiolar gradient of voltage-dependent AZ activation (i.e., stronger AZs on the pillar side of the cell in terms of V_h_) was previously demonstrated for WT IHCs (Ohn et al., 2016; Özçete and Moser, 2021). Correlation analysis of V_h_, ΔF/F_0max_ and synapse position revealed weak but significant positive correlations (Supplementary Table 1). These are consistent with the notion that modiolar synapses of Vglut3^+/+^ and Vglut3^–/–^ IHCs have stronger maximal synaptic Ca^2 +^ influx (larger ΔF/F_0max_) yet activating at more depolarized potentials (larger V_h_). Together, these results indicate mild shift of AZ activation to lower voltages but intact spatial gradients of the maximal amplitude and the voltage of half maximal activation of synaptic Ca^2+^ influx in *Vglut3*^–/–^ IHCs.

### 3.2. Structural heterogeneity of Ca_*v*_1.3 channel clusters and synaptic ribbons in IHCs of *Vglut3*^–/–^ mice

**FIGURE 4:**
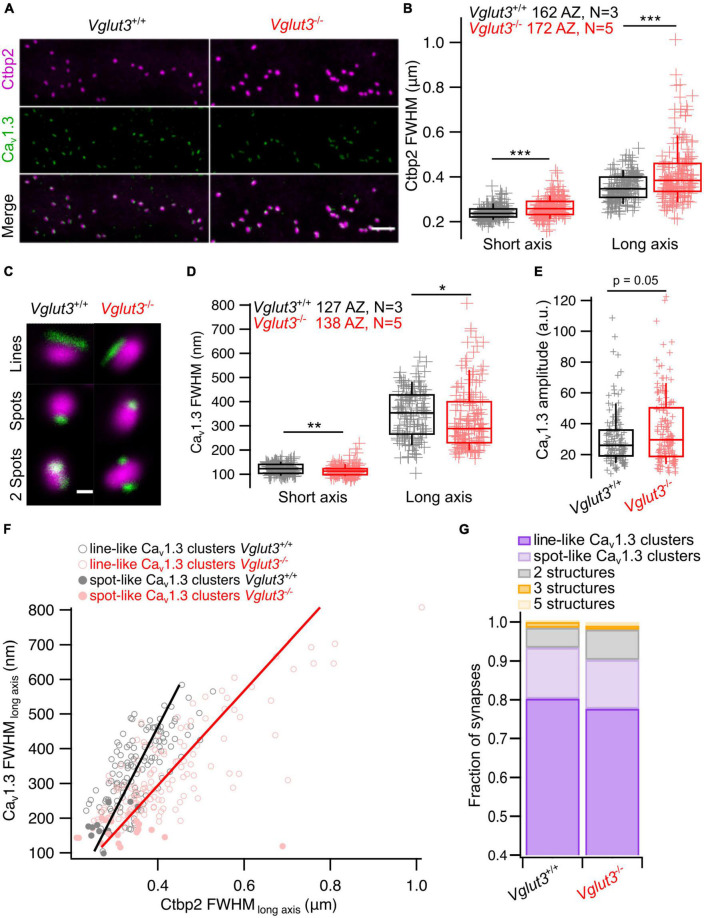
Analyzing synaptic ribbons and Ca_v_1.3 channel clusters using 2D STED microscopy. **(A)** Representative maximum intensity projections of confocal stacks immunostained for Ctbp2 and Ca_v_1.3. Scale bar = 3 μm. **(B)** FWHM of short and long axes obtained by fitting 2D STED images of Ctbp2 immunofluorescent spots with 2D Gaussian function show enlarged ribbon size in IHCs of *Vglut3*^–/–^ mice when comparing FWHM of both short (*Vglut3^+/+^*: 241 ± 2.4 nm, SD = 30 nm, 162 AZs; *Vglut3*^–/–^: 263 ± 3.4 nm, SD = 45 nm, 172 AZs; Mann-Whitney-Wilcoxon test, *p* < 0.001) and long (*Vglut3^+/+^*: 353 ± 4.7 nm, SD = 60 nm, 162 AZs; *Vglut3*^–/–^: 415 ± 10 nm, SD = 131 nm, 172 AZs; Mann-Whitney-Wilcoxon test, *p* < 0.001) axes. **(C)** Representative 2D STED images of ribbons colocalized with most prevalent types of Ca_v_1.3 clusters. **(D)** Ca_v_1.3 line-like clusters fitted with 2D Gaussian function show decreased FWHM of short (*Vglut3^+/+^*: 122 ± 1.94 nm, SD = 21.8 nm, 127 AZs; *Vglut3*^–/–^: 114 ± 1.98 nm, SD = 23.3 nm, 138 AZs; Mann-Whitney-Wilcoxon test, *p* = 0.002) and long (*Vglut3^+/+^*: 349 ± 8.9 nm, SD = 101 nm, 127 AZs; *Vglut3*^–/–^: 331 ± 11.2 nm, SD = 131 nm, 138 AZs; Mann-Whitney-Wilcoxon test, *p* = 0.015) axes in Vglut3 KO IHCs. **(E)** Ca_v_1.3 immunofluorescence amplitude obtained from 2D Gaussian fits of line- and spot-like clusters is higher in Vglut3 KO IHCs (*Vglut3^+/+^*: 30.3 ± 1.47, SD = 17.4, 141 AZs; *Vglut3*^–/–^: 36.3 ± 1.79, SD = 22.6, 158 AZs; Mann-Whitney-Wilcoxon test, *p* = 0.05). **(F)** Relationship between Ca_v_1.3 and Ctbp2 FWHM of long axes (*Vglut3^+/+^*: P_r_ = 0.73; *Vglut3*^–/–^: P_r_ = 0.78). **(G)** Ca_v_1.3 clusters in WT and Vglut3 KO IHCs were categorized into line-like clusters, spot-like clusters, 2, 3, 5 structures containing clusters. Ca_v_1.3 cluster categories were not changed in Vglut3 KO IHCs. Box-whisker plots with individual data points overlaid show median (middle line), 25^th^ and 75^th^ percentiles (lower and upper borders of the box) and 10^th^ and 90^th^ percentiles (whiskers). Asterisks indicate statistical significance (*p < 0.05, **p < 0.01, ***p < 0.001).

While the heterogeneity of Ca^2+^ influx among IHCs was largely preserved in *Vglut3*^–/–^ IHCs, diversity of AZ morphology could still be affected. To test this, we first performed 2D STED nanoscopy at AZs which were immunolabeling against Ctbp2/RIBEYE and Ca_*v*_1.3 to examine the morphology of ribbons and Ca_*V*_1.3 clusters (Krinner et al., 2017; Jean et al., 2018, 2019; Neef et al., 2018, Figure 4A). First, we fitted Ctbp2 immunofluorescent spots with a 2D Gaussian function and observed increased full widths at half maxima (FWHM) of both long and short axes in *Vglut3*^–/–^ IHCs (Figures 4B, C). This abnormal enlargement or elongation of the ribbons in IHCs of knockout or mutated Vglut3 mice has been observed in previous studies (Seal et al., 2008; Kim et al., 2019; Joshi et al., 2021). *Vglut3*^+/−^ animals did not show significant difference in FWHM of long axis of Ctbp2 immunofluorescent spots (*Vglut3*^+/+^: 378 ± 5.7 nm, SD = 55 nm, *n* = 92, *N* = 2 vs *Vglut3*^+/−^: 380 ± 9.3 nm, SD = 62 nm, *n* = 44, *N* = 3, Student’s *t*-test, *p* = 0.87). We found a mild but significant increase in the FWHM of short axis in *Vglut3*^+/−^ animals (*Vglut3*^+/+^: 250 ± 3.3 nm, SD = 32 nm, *n* = 92, *N* = 2 vs *Vglut3*^+/−^: 261 ± 4.6 nm, SD = 31 nm, *n* = 44, *N* = 3, Mann-Whitney-Wilcoxon test, *p* = 0.015). In total, the area of the ribbons (pi*(FWHM_long_axis/2) *(FWHM_short_axis)) in *Vglut3*^+/−^ animals was not significantly changed, consistent with the previous report (Kim et al., 2019). Next, we classified the different morphologies of Ca_*v*_1.3 clusters (Krinner et al., 2017; Jean et al., 2018, 2019; Neef et al., 2018). We did not observe any abnormalities in the proportions of the cluster types in *Vglut3*^–/–^ IHCs: the majority of the Ca_*v*_1.3 clusters were line- (around 80%) or spot-like (over 10%) in both WT and *Vglut3*^–/–^ IHCs (Figure 4G). By fitting the 2D Gaussian function to the population of line-like Ca_*v*_1.3 clusters of WT and *Vglut3*^–/–^ IHCs, we saw a reduction in FWHM of both long and short axes of IHC Ca_*v*_1.3 clusters in *Vglut*^–/–^ mice (Figures 4D, F). However, this was not reflected in a reduction of amplitude of the 2D Gaussian fit: instead, Ca_*v*_1.3 clusters of *Vglut3*^–/–^ IHCs showed a trend toward an increased amplitude of the 2D Gaussian fit that did not reach significance (Figure 4E). A tighter spatial confinement of a greater number of Ca_*v*_1.3 channels at AZs of *Vglut3*^–/–^ IHCs could offer a potential explanation for our findings from synaptic Ca^2+^ imaging and STED nanoscopy.

**FIGURE 5:**
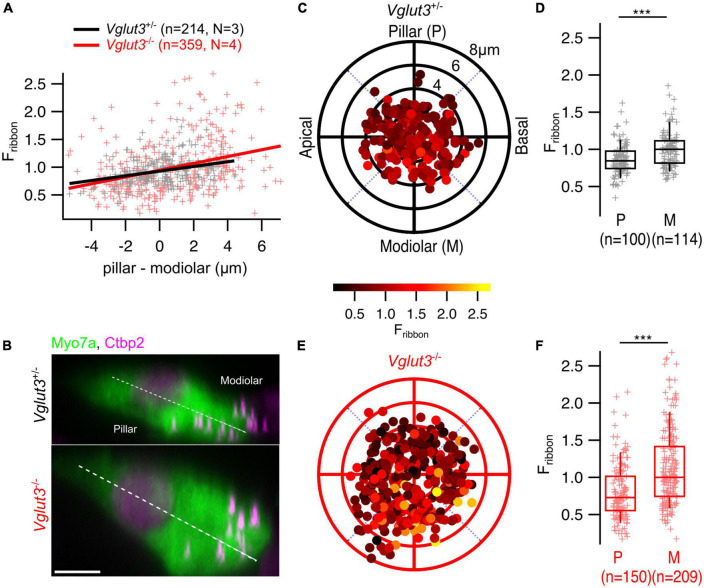
The spatial gradient of ribbon size is preserved in IHCs of *Vglut3*^–/–^ mice. **(A)** Ribbon intensity as a function of its position along the modiolar-pillar axis of the IHC. Solid lines show the linear regression. **(B)** Maximum intensity projections from representative IHCs in YZ direction show brighter ribbons on the modiolar side of the cell in both *Vglut3^+/–^* and *Vglut3*^–/–^ IHCs. Scale bar = 5 μm. Polar plots show the locations of individual AZs in *Vglut3^+/–^* **(C)** and *Vglut3*^–/–^ **(E)** IHCs. Pseudo-color scale shows normalized Ctbp2 immunofluorescence intensity (F_ribbon_). Ctbp2 intensity was normalized to the median intensity of the modiolar ribbons for each sample. **(D,F)** Box plots compare F_ribbon_ of pillar and modiolar ribbons and show stronger signal at modiolar AZs compared to the pillar AZs in both *Vglut3^+/–^* (pillar: 0.86 ± 0.02, SD = 0.22, 100 spots; modiolar: 0.99 ± 0.02, SD = 0.24, 114 spots; Mann-Whitney-Wilcoxon test, *p* < 0.0001) and *Vglut3*^–/–^ (pillar: 0.81 ± 0.03, SD = 0.38, 150 spots; modiolar: 1.1 ± 0.04, SD = 0.51, 209 spots; Mann-Whitney-Wilcoxon test, *p* < 0.0001) IHCs. Box-whisker plots with individual data points overlaid show median (middle line), 25^th^ and 75^th^ percentiles (lower and upper borders of the box) and 10^th^ and 90^th^ percentiles (whiskers). Asterisks indicate statistical significance (***p < 0.001).

Next, we tested for spatial gradients of ribbon size, approximated as immunofluorescent intensity in confocal stacks of IHCs (Ohn et al., 2016; Jean et al., 2019; Sherrill et al., 2019) immunostained for Myo7a and Ctbp2/RIBEYE (Figure 5B). For these experiments, we used *Vglut3^+/–^* mice as controls. Overall, we found that the modiolar-pillar gradient of ribbon size (i.e., larger AZs on the modiolar side of the cell in terms of ribbon size) is maintained in *Vglut3*^–/–^ IHCs (*Vglut3^+/–^*: pillar: 0.86 ± 0.02, SD = 0.22, 100 spots; modiolar: 0.99 ± 0.02, SD = 0.24, 114 spots; Mann-Whitney-Wilcoxon test, *p* < 0.0001; *Vglut3*^–/–^: pillar: 0.81 ± 0.03, SD = 0.38, 150 spots; modiolar: 1.1 ± 0.04, SD = 0.51, 209 spots; Mann-Whitney-Wilcoxon test, *p* < 0.0001; Figures 5A, C–F).

### 3.3. Heterogeneity of Ca^2+^ influx at the AZs of *Otof^TDA/TDA^* IHCs

Our analysis of *Vglut3*^–/–^ mice showed intact modiolar-pillar gradients of maximal synaptic Ca^2+^ influx and ribbon size in IHCs. Moreover, despite a mild overall hyperpolarized shift of the voltage of half-maximal Ca^2+^ channel activation, its pillar-modiolar gradient was preserved in *Vglut3*^–/–^ IHCs. This argues against a strict requirement of glutamatergic signaling for establishing or maintaining IHC synaptic heterogeneity. To test the impact of evoked exocytosis on the AZ heterogeneity and the spatial gradients of AZ properties, we chose to study a novel *Otof* mutant mouse. These mice harbor a triple aspartate to alanine mutation in the C_2_E domain of otoferlin *Otof^TDA/TDA^* —a protein that has been shown to be vital for IHC vesicle fusion (Roux et al., 2006; Pangrsic et al., 2010; Michalski et al., 2017). Exocytosis in IHCs of *Otof^TDA/TDA^* is largely abolished, but, unlike in other *Otof* mutants (e.g. 50% synapse loss in IHCs of *Otof*^–/–^ mice, Roux et al., 2006; Stalmann et al., 2021) the number of the IHC afferent synapses was preserved in 2–3-week-old mice (Chen et al.).

**FIGURE 6:**
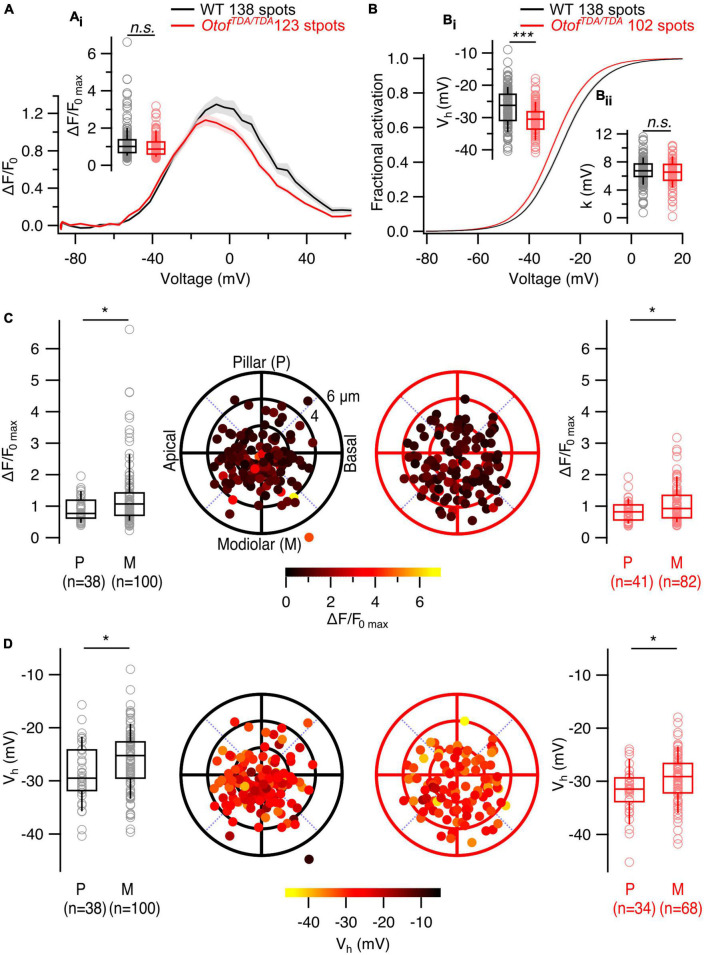
The spatial gradients of Ca^2+^ influx properties are not altered in IHCs of *Otof^TDA/TDA^* mice. **(A)** Average ΔF/F_0_ traces of single AZs plotted against depolarization voltages. Shaded areas represent ± SEM. **(A_i_)** Box plot shows no difference in single synaptic maximum Ca^2+^ influx between WT and *Otof^TDA/TDA^* IHCs (WT: 1.22 ± 0.079, SD = 0.92, 138 spots, *n* = 30, *N* = 17; *Otof^TDA/TDA^*: 1.00 ± 0.048, SD = 0.53, 123 spots, *n* = 13, *N* = 7; Mann-Whitney-Wilcoxon test, *p* = 0.07). **(B)** Average fractional activation curves of single AZs in WT and *Otof^TDA/TDA^* IHCs. Shaded areas represent ± SEM. **(B_i_)** Box plots comparing voltage of half maximal activation (V_h_) at single AZ level show significant hyperpolarized shift in IHCs of *Otof^TDA/TDA^* mice (WT: –26.8 ± 0.49 mV, SD = 5.7 mV, 138 spots, *n* = 30, *N* = 17 *Otof^TDA/TDA^*: –30 ± 0.47 mV, SD = 4.78 mV, 102 spots, *n* = 13, *N* = 7; Student’s *t*-test, *p* < 0.0001). **(B_ii_)** Voltage sensitivity of Ca^2+^ influx at single AZ level shows no difference between IHCs of WT and *Otof^TDA/TDA^* mice (WT: 6.77 ± 0.15 mV, SD = 1.75 mV, 138 spots, *n* = 30, *N* = 17; *Otof^TDA/TDA^*: 6.32 ± 0.19 mV, SD = 1.87 mV, 102 spots, *n* = 13, *N* = 7; Student’s *t*-test, *p* = 0.06). **(C)** Polar plots show the locations of individual AZs in WT (left, black) and *Otof^TDA/TDA^* (right, red) IHCs. Pseudo-color scale shows ΔF/F_0 max_. Box plots compare ΔF/F_0 max_ of pillar and modiolar AZs and show larger Ca^2+^ influx at modiolar AZs compared to the pillar AZs in both WT (pillar: 0.9 ± 0.06, SD = 0.38, 38 spots; modiolar: 1.3 ± 0.1, SD = 1.03, 100 spots; Mann-Whitney-Wilcoxon test, *p* = 0.02) and *Otof^TDA/TDA^* (pillar: 0.84 ± 0.05, SD = 0.33, 41 spots; modiolar: 1.08 ± 0.07, SD = 0.6, 82 spots; Mann-Whitney-Wilcoxon test, *p* = 0.04) IHCs. **(D)** Polar plots show the locations of individual AZs in WT (left, black) and *Otof^TDA/TDA^* (right, red) IHCs. Pseudo-color scale shows voltage of half maximal activation. Box plots compare V_h_ of pillar and modiolar AZs and show more depolarized V_h_ at modiolar AZs compared to the pillar AZs in both WT (pillar: –28.8 ± 0.91 mV, SD = 5.61 mV, 38 spots; modiolar: –26 ± 0.57 mV, SD = 5.65 mV, 100 spots; Student’s *t*-test, *p* = 0.01) and *Otof^TDA/TDA^* (pillar: –31.8 ± 0.75 mV, SD = 4.38 mV, 34 spots; modiolar: –29.5 ± 0.58 mV, SD = 4.81 mV, 68 spots; Mann-Whitney-Wilcoxon test, *p* = 0.01) IHCs.

Overall, *Otof^TDA/TDA^* mice allow us to analyze the presynaptic heterogeneity in IHCs largely lacking exocytosis while avoiding any sampling bias due to synaptic degeneration. We performed patch-clamp combined with single AZ Ca^2+^ imaging from the IHCs of P21-P28 *Otof^TDA/TDA^* mice similar to the Vglut3^–/–^ mice. We observed a non-significant trend toward lower maximal amplitude of synaptic Ca^2+^ influx (WT: 1.22 ± 0.079, SD = 0.92, 138 spots, *n* = 30, *N* = 17; *Otof^TDA/TDA^*: 1.00 ± 0.048, SD = 0.53, 123 spots, *n* = 13, *N* = 7; Mann-Whitney-Wilcoxon test, *p* = 0.07; Figures 6A, A_i_). The modiolar-pillar gradient of the maximum synaptic Ca^2+^ influx was not affected (pillar: 0.84 ± 0.05, SD = 0.33, 41 spots; modiolar: 1.08 ± 0.07, SD = 0.6, 82 spots; Mann-Whitney-Wilcoxon test, *p* = 0.04; Figure 6C). Analysis of the voltage dependence of Ca^2+^ channel activation revealed a significant hyperpolarized shift of V_*h*_ in *Otof^TDA/TDA^* mice (WT: −26.8 ± 0.49 mV, SD = 5.7 mV, 138 spots, *n* = 30, *N* = 17; *Otof^TDA/TDA^*: −30 ± 0.47 mV, SD = 4.78 mV, 102 spots, *n* = 13, *N* = 7; Student’s *t*-test, *p* < 0.0001; Figures 6B, B_i_) but no change was observed in the voltage sensitivity of channel activation (WT: 6.77 ± 0.15 mV, SD = 1.75 mV, 138 spots, *n* = 30, *N* = 17; *Otof*^*TDA*/*TDA*^: 6.32 ± 0.19 mV, SD = 1.87 mV, 102 spots, *n* = 13, *N* = 7; Student’s *t*-test, *p* = 0.06; Figure 6B_ii_) but no change was observed in the voltage sensitivity of channel activation (WT: 6.77 ± 0.15 mV, SD = 1.75 mV, 138 spots, *n* = 30, *N* = 17; *Otof*^*TDA*/*TDA*^: 6.32 ± 0.19 mV, SD = 1.87 mV, 102 spots, *n* = 13, *N* = 7; Student’s *t*-test, *p* = 0.06; Figure 6B_ii_). Despite this shift, the pillar-modiolar gradient of V_h_ was preserved, with pillar AZs activating at lower voltage than modiolar AZs (pillar: −31.8 ± 0.75 mV, SD = 4.38 mV, 34 spots; modiolar: −29.5 ± 0.58 mV, SD = 4.81 mV, 68 spots; Mann-Whitney-Wilcoxon test, *p* = 0.01; Figure 6D). Interestingly, the distributions of V_h_ in WT and *Otof^TDA/TDA^* IHCs differ also in the variance (Supplementary Figure 2). Interquartile ranges of the two V_h_ distributions suggest narrower spread of V_h_ in *Otof^TDA/TDA^* mice (WT: 8.14 mV vs. *Otof^TDA/TDA^*: 5.38 mV). Consistent with that observation, Levene’s test showed a significant difference between the two distributions, indicating inequality of variances.

## 4. Discussion

Heterogeneity of IHC afferent synapses is one of the candidate mechanisms contributing to the functional diversity of type I SGNs in the cochlea. In this study we aimed to understand the role of synaptic transmission in the establishment of AZ heterogeneity in IHCs. For this reason, we employed two mouse models: *Vglut3*^–/–^ mice, which lack glutamatergic signaling at IHC afferent synapses, but maintain vesicle fusion, and *Otof^TDA/TDA^* mice, IHCs of which display largely abolished evoked exocytosis. By Ca^2+^ imaging, we analyzed the amplitude of synaptic Ca^2+^ influx and its voltage dependence of activation at single AZs of IHCs in *Vglut3*^–/–^ and *Otof^TDA/TDA^* mutant mice. We then related those properties to the positions of the AZs along the modiolar-pillar axis of mutant IHCs and did not observe any striking changes of the previously described gradients (Ohn et al., 2016). Additionally, the modiolar-pillar gradient of ribbon size was retained in IHCs of *Vglut3*^–/–^ mice. We conclude that synaptic transmission at IHC afferent synapses is dispensable for the formation of presynaptic AZ heterogeneity. However, we observed an overall hyperpolarized shift of Ca^2+^ channel activation in IHCs of both mutants and a lower variance of the voltage dependence of Ca^2+^ channel activation in *Otof^TDA/TDA^* IHCs.

Spiral ganglion neuron molecular subtypes in mice are established already at birth (Petitpré et al., 2018). Glutamatergic synaptic transmission during cochlear development is crucial for maintaining proper type I SGN molecular subtype specification (Shrestha et al., 2018; Sun et al., 2018). Specifically, transcriptomic studies have shown that disrupting glutamatergic transmission in *Vglut3*^–/–^ mice reduces the number of type I_b_ and I_c_ SGNs, and the majority of neurons gain a type I_a_ fate. RNAscope results from *Vglut3*^–/–^ mouse tissues do not show obvious deviations from the WT mice at postnatal day P3, and the alterations in SGN molecular subtypes start between postnatal day 3 and 8 (Shrestha et al., 2018). In return, postsynaptic SGNs seem to regulate AZ properties by transsynaptic signaling. For example, expression of the transcription factor *Pou4f1* is characteristic for type I_c_ and I_b_ SGNs and contributes to shaping AZ properties. Conditional deletion of *Pou4f1* disrupted the modiolar-pillar gradient of the maximal amplitude of synaptic Ca^2+^ influx in IHCs (Sherrill et al., 2019). Similarly, deletion of the *Runx1* transcription factor that is selectively expressed in type I_c_ and I_b_ SGNs in a recent study resulted in a collapse of the modiolar-pillar gradient of ribbon size in IHCs (Shrestha et al., 2023). We hypothesized similar changes in *Vglut3*^–/–^ mice considering the dramatic reduction of type I_b_ and I_c_ neurons. Interestingly, we observed intact gradients of maximal Ca^2+^ influx, voltage dependence of Ca^2+^ channels, and ribbon size of AZs along the modiolar-pillar axis. This might suggest a limited impact of transsynaptic signaling for IHC AZ heterogeneity. Alternatively, transsynaptic signaling might be maintained despite the altered molecular identity or largely restricted to a certain time window during the early postnatal development of the animal. The latter might explain the discrepancy observed upon postnatal disruption of SGN subtype specification in *Vglut3*^–/–^ mice to the findings upon perinatal conditional *Pou4f1* deletion. Interestingly, we also observed an intact modiolar-pillar gradient of maximal Ca^2+^ influx for exocytosis-deficient AZs of *Otof* mutant mice for which the molecular subtype specification of SGN remains to be studied. *Otof* mutation would be expected to spare early synaptic signaling as IHC exocytosis in mice is independent of otoferlin during the first 3 postnatal days (Beurg et al., 2010). Finally, we observed an intact modiolar-pillar gradient of maximal Ca^2+^ influx in *dfcr* mutants (Ohn et al., 2016) with disrupted function of the Usher protein harmonin that is required for normal mechanoelectrical transduction (Grillet et al., 2009; Michalski et al., 2009) and also regulates synaptic Ca^2+^ influx (Gregory et al., 2011).

All four manipulations, i.e., disruption of the function of harmonin, otoferlin, Vglut3, and Pou4F1, resulted in a mild hyperpolarized shift of Ca^2+^ channel activation at IHC AZs. Considering the reduced fraction of SGNs with type I_b/c_ identity in *Pou4F1 and Vglut3* mutant mice one might speculate on a longer-term requirement of type I_b_ and I_*c*_-specific transsynaptic signaling at IHC afferent synapses. However, other mechanisms might contribute to the change in Ca^2+^ channel activation. This seems likely at least for disruption of the function of harmonin and otoferlin, which both interact with Ca_v_1.3 channels (Ramakrishnan et al., 2009; Gregory et al., 2011).

### 4.1. Limitations of the present study

We note that all the experiments in this study have been performed at the apical turn of the organ of Corti around the 6–8 kHz region. Therefore, we cannot exclude the possibility that larger effects of impaired transsynaptic signaling could be observed at other tonotopic positions. Furthermore, despite the preserved gradients of IHC AZ properties, *Vglut3*^–/–^ animals display abnormal enlargement of synaptic ribbons. These results are inconsistent with the findings in Pou4f1 and Runx1 KOs, where deletion of type I_b_ and I_c_ SGN molecular markers led to reduction of average ribbon size. Thus, there is the possibility that direct presynaptic alterations upon Vglut3 deletion mask or overrule potential effects of the altered SGN molecular subtype specification.

## Data availability statement

The original contributions presented in this study are included in the article/Supplementary material, further inquiries can be directed to the corresponding author.

## Ethics statement

The animal study was approved by the local Animal Welfare Committee of the University Medical Center Göttingen and the Max Planck Institute for Multidisciplinary Sciences, as well as the Animal Welfare Office of the state of Lower Saxony, Germany (LAVES, AZ 19/3134). The study was conducted in accordance with the local legislation and institutional requirements.

## Author contributions

NK performed the experiments and data analysis. Both authors designed the study and prepared the manuscript.

## References

[B1] Becker L. Schnee M. Niwa M. Sun W. Maxeiner S. Talaei S. (2018). The presynaptic ribbon maintains vesicle populations at the hair cell afferent fiber synapse. *eLife* 7:e30241. 10.7554/eLife.30241 29328021 PMC5794257

[B2] Beurg M. Michalski N. Safieddine S. Bouleau Y. Schneggenburger R. Chapman E. (2010). Control of exocytosis by synaptotagmins and otoferlin in auditory hair cells. *J. Neurosci.* 30 13281–13290. 10.1523/JNEUROSCI.2528-10.2010 20926654 PMC3088501

[B3] Cantu-Guerra H. Papazian M. Gorsky A. Alekos N. Caccavano A. Karagulyan N. (2023). Cochlear hair cell innervation is dependent on a modulatory function of semaphorin-3A. *Dev. Dyn.* 252 124–144. 10.1002/dvdy.548 36284453 PMC9812910

[B4] Chen H., Monga M., Fang Q., Slitin L., Neef J. Ca^2+^-binding to the C2E and C2F domain of otoferlin is required for hair cell exocytosis and hearing..

[B5] Frank T. Khimich D. Neef A. Moser T. (2009). Mechanisms contributing to synaptic Ca2+ signals and their heterogeneity in hair cells. *Proc. Natl. Acad. Sci. U. S. A.* 106 4483–4488. 10.1073/pnas.0813213106 19246382 PMC2657422

[B6] Frank T. Rutherford M. Strenzke N. Neef A. Pangršič T. Khimich D. (2010). Bassoon and the synaptic ribbon organize Ca^2 +^ channels and vesicles to add release sites and promote refilling. *Neuron* 68 724–738. 10.1016/j.neuron.2010.10.027 21092861 PMC3005353

[B7] Gregory F. Bryan K. Pangršič T. Calin-Jageman I. Moser T. Lee A. (2011). Harmonin inhibits presynaptic Cav1.3 Ca^2 +^ channels in mouse inner hair cells. *Nat. Neurosci.* 14 1109–1111. 10.1038/nn.2895 21822269 PMC3164920

[B8] Grillet N. Xiong W. Reynolds A. Kazmierczak P. Sato T. Lillo C. (2009). Harmonin mutations cause mechanotransduction defects in cochlear hair cells. *Neuron* 62 375–387. 10.1016/j.neuron.2009.04.006 19447093 PMC2691393

[B9] Hickman T. Liberman M. Jacob M. (2015). Adenomatous polyposis coli protein deletion in efferent olivocochlear neurons perturbs afferent synaptic maturation and reduces the dynamic range of hearing. *J. Neurosci.* 35 9236–9245. 10.1523/JNEUROSCI.4384-14.2015 26085645 PMC4469744

[B10] Hua Y. Ding X. Wang H. Wang F. Lu Y. Neef J. (2021). Electron microscopic reconstruction of neural circuitry in the cochlea. *Cell Rep.* 34 108551. 10.1016/j.celrep.2020.108551 33406431

[B11] Jean P. Lopez de la Morena D. Michanski S. Jaime Tobón L. M. Chakrabarti R. Picher M. M. (2018). The synaptic ribbon is critical for sound encoding at high rates and with temporal precision. *eLife* 7:e29275. 10.7554/eLife.29275 29328020 PMC5794258

[B12] Jean P. Özçete Ö Tarchini B. Moser T. (2019). Intrinsic planar polarity mechanisms influence the position-dependent regulation of synapse properties in inner hair cells. *Proc. Natl. Acad. Sci. U. S. A.* 116 9084–9093. 10.1073/pnas.1818358116 30975754 PMC6500111

[B13] Joshi Y. Petit C. Miot S. Guillet M. Sendin G. Bourien J. (2021). VGLUT3-p.A211V variant fuses stereocilia bundles and elongates synaptic ribbons. *J. Physiol.* 599 5397–5416. 10.1113/JP282181 34783032 PMC9299590

[B14] Jung S. Oshima-Takago T. Chakrabarti R. Wong A. Jing Z. Yamanbaeva G. (2015). Rab3-interacting molecules 2α and 2β promote the abundance of voltage-gated CaV1.3 Ca2+ channels at hair cell active zones. *Proc. Natl. Acad. Sci. U. S. A.* 112 E3141–E3149. 10.1073/pnas.1417207112 26034270 PMC4475996

[B15] Kiang N. Y. S. Watanabe T. Thomas E. C. Clark L. F. (1965). *Discharge patterns of single fibers in the cat’s auditory nerve.* Cambridge, MA: MIT Press.

[B16] Kim K. Payne S. Yang-Hood A. Li S. Davis B. Carlquist J. (2019). Vesicular glutamatergic transmission in noise-induced loss and repair of cochlear ribbon synapses. *J. Neurosci.* 39 4434–4447. 10.1523/JNEUROSCI.2228-18.2019 30926748 PMC6554621

[B17] Krinner S. Butola T. Jung S. Wichmann C. Moser T. (2017). RIM-binding protein 2 promotes a large number of CaV1.3 Ca2+-channels and contributes to fast synaptic vesicle replenishment at hair cell active zones. *Front. Cell Neurosci.* 11:334. 10.3389/fncel.2017.00334 29163046 PMC5673845

[B18] Krinner S. Predoehl F. Burfeind D. Vogl C. Moser T. (2021). RIM-binding proteins are required for normal sound-encoding at afferent inner hair cell synapses. *Front. Mol. Neurosci.* 14:651935. 10.3389/fnmol.2021.651935 33867935 PMC8044855

[B19] Li C. Li X. Bi Z. Sugino K. Wang G. Zhu T. (2020). Comprehensive transcriptome analysis of cochlear spiral ganglion neurons at multiple ages. *eLife* 9:e50491. 10.7554/eLife.50491 31913118 PMC7299348

[B20] Liberman L. Wang H. Liberman M. (2011). Opposing gradients of ribbon size and AMPA receptor expression underlie sensitivity differences among cochlear-nerve/hair-cell synapses. *J. Neurosci.* 31 801–808. 10.1523/JNEUROSCI.3389-10.2011 21248103 PMC3290333

[B21] Liberman M. (1978). Auditory-nerve response from cats raised in a low-noise chamber. *J. Acoust. Soc. Am.* 63 442–455. 10.1121/1.381736 670542

[B22] Liberman M. (1982). Single-neuron labeling in the cat auditory nerve. *Science* 216 1239–1241. 10.1126/science.7079757 7079757

[B23] Merchan-Perez A. Liberman M. (1996). Ultrastructural differences among afferent synapses on cochlear hair cells: correlations with spontaneous discharge rate. *J. Comp. Neurol.* 371 208–221. 8835727 10.1002/(SICI)1096-9861(19960722)371:2<208::AID-CNE2>3.0.CO;2-6

[B24] Meyer A. Frank T. Khimich D. Hoch G. Riedel D. Chapochnikov N. (2009). Tuning of synapse number, structure and function in the cochlea. *Nat. Neurosci.* 12 444–453. 10.1038/nn.2293 19270686

[B25] Meyer A. Moser T. (2010). Structure and function of cochlear afferent innervation. *Curr. Opin. Otolaryngol. Head Neck. Surg.* 18 441–446. 10.1097/MOO.0b013e32833e0586 20802334

[B26] Michalski N. Goutman J. Auclair S. Boutet de Monvel J. Tertrais M. Emptoz A. (2017). Otoferlin acts as a Ca2+ sensor for vesicle fusion and vesicle pool replenishment at auditory hair cell ribbon synapses. *eLife* 6:e31013. 10.7554/eLife.31013 29111973 PMC5700815

[B27] Michalski N. Michel V. Caberlotto E. Lefèvre G. van Aken A. Tinevez J. (2009). Harmonin-b, an actin-binding scaffold protein, is involved in the adaptation of mechanoelectrical transduction by sensory hair cells. *Pflugers Arch.* 459 115–130. 10.1007/s00424-009-0711-x 19756723 PMC2767239

[B28] Neef J. Urban N. Ohn T. Frank T. Jean P. Hell S. (2018). Quantitative optical nanophysiology of Ca^2 +^ signaling at inner hair cell active zones. *Nat. Commun.* 9 290. 10.1038/s41467-017-02612-y 29348575 PMC5773603

[B29] Ohn T. Rutherford M. Jing Z. Jung S. Duque-Afonso C. Hoch G. (2016). Hair cells use active zones with different voltage dependence of Ca^2 +^ influx to decompose sounds into complementary neural codes. *Proc. Natl. Acad. Sci. U. S. A.* 113 E4716–E4725. 10.1073/pnas.1605737113 27462107 PMC4987782

[B30] Özçete Ö Moser T. (2021). A sensory cell diversifies its output by varying Ca^2 +^ influx-release coupling among active zones. *EMBO J.* 40:e106010. 10.15252/embj.2020106010 33346936 PMC7917556

[B31] Pangrsic T. Lasarow L. Reuter K. Takago H. Schwander M. Riedel D. (2010). Hearing requires otoferlin-dependent efficient replenishment of synaptic vesicles in hair cells. *Nat. Neurosci.* 13 869–876. 10.1038/nn.2578 20562868

[B32] Petitpré C. Wu H. Sharma A. Tokarska A. Fontanet P. Wang Y. (2018). Neuronal heterogeneity and stereotyped connectivity in the auditory afferent system. *Nat. Commun.* 9 3691. 10.1038/s41467-018-06033-3 30209249 PMC6135759

[B33] Ramakrishnan N. Drescher M. Drescher D. (2009). Direct interaction of otoferlin with syntaxin 1A, SNAP-25, and the L-type voltage-gated calcium channel Cav1.3. *J. Biol. Chem.* 284 1364–1372. 10.1074/jbc.M803605200 19004828 PMC2615516

[B34] Roux I. Safieddine S. Nouvian R. Grati M. Simmler M. Bahloul A. (2006). Otoferlin, defective in a human deafness form, is essential for exocytosis at the auditory ribbon synapse. *Cell* 127 277–289. 10.1016/j.cell.2006.08.040 17055430

[B35] Ruel J. Emery S. Nouvian R. Bersot T. Amilhon B. Van Rybroek J. (2008). Impairment of SLC17A8 encoding vesicular glutamate transporter-3, VGLUT3, underlies nonsyndromic deafness DFNA25 and inner hair cell dysfunction in null mice. *Am. J. Hum. Genet.* 83 278–292. 10.1016/j.ajhg.2008.07.008 18674745 PMC2495073

[B36] Sachs M. Abbas P. (1974). Rate versus level functions for auditory-nerve fibers in cats: tone-burst stimuli. *J. Acoust. Soc. Am.* 56 1835–1847. 10.1121/1.1903521 4443483

[B37] Sanchez del Rio M. Pareschi G. (2001). “Global Optimization and Reflectivity Data Fitting for X-Ray Multilayer Mirrors by Means of Genetic Algorithms,” in *SPIE Proceedings*, Basel, 10.1117/12.411624

[B38] Seal R. Akil O. Yi E. Weber C. Grant L. Yoo J. (2008). Sensorineural deafness and seizures in mice lacking vesicular glutamate transporter 3. *Neuron* 57 263–275. 10.1016/j.neuron.2007.11.032 18215623 PMC2293283

[B39] Sherrill H. Jean P. Driver E. Sanders T. Fitzgerald T. Moser T. (2019). Pou4f1 defines a subgroup of type I spiral ganglion neurons and is necessary for normal inner hair cell presynaptic Ca2+ signaling. *J. Neurosci.* 39 5284–5298. 10.1523/JNEUROSCI.2728-18.2019 31085606 PMC6607758

[B40] Shrestha B. Chia C. Wu L. Kujawa S. Liberman M. Goodrich L. (2018). Sensory neuron diversity in the inner ear is shaped by activity. *Cell* 174 1229–1246.e17. 10.1016/j.cell.2018.07.007 30078709 PMC6150604

[B41] Shrestha B. Wu L. Goodrich L. (2023). Runx1 controls auditory sensory neuron diversity in mice. *Dev. Cell* 58 306–319.e5. 10.1016/j.devcel.2023.01.008 36800995 PMC10202259

[B42] Siebald C. Vincent P. F. Y. Bottom R. T. Sun S. Reijntjes D. O. J. Manca M. (2023). Molecular signatures define subtypes of auditory afferents with distinct peripheral projection patterns and physiological properties. *Proc. Natl. Acad. Sci. U.S.A*. 120:e2217033120. 10.1073/pnas.2217033120 37487063 PMC10400978

[B43] Stalmann U. Franke A. Al-Moyed H. Strenzke N. Reisinger E. (2021). Otoferlin is required for proper synapse maturation and for maintenance of inner and outer hair cells in mouse models for DFNB9. *Front. Cell Neurosci.* 15:677543. 10.3389/fncel.2021.677543 34335185 PMC8316924

[B44] Sun S. Babola T. Pregernig G. So K. Nguyen M. Su S. (2018). Hair cell mechanotransduction regulates spontaneous activity and spiral ganglion subtype specification in the auditory system. *Cell* 174 1247–1263.e15. 10.1016/j.cell.2018.07.008 30078710 PMC6429032

[B45] Winter I. Robertson D. Yates G. (1990). Diversity of characteristic frequency rate-intensity functions in guinea pig auditory nerve fibres. *Hear Res.* 45 191–202. 10.1016/0378-5955(90)90120-e 2358413

[B46] Yin Y. Liberman L. Maison S. Liberman M. (2014). Olivocochlear innervation maintains the normal modiolar-pillar and habenular-cuticular gradients in cochlear synaptic morphology. *J. Assoc. Res. Otolaryngol.* 15 571–583. 10.1007/s10162-014-0462-z 24825663 PMC4141434

[B47] Zenisek D. Horst N. Merrifield C. Sterling P. Matthews G. (2004). Visualizing synaptic ribbons in the living cell. *J. Neurosci.* 24 9752–9759. 10.1523/JNEUROSCI.2886-04.2004 15525760 PMC6730242

