# Supplementary material for: Synaptic activity is not required for establishing heterogeneity of inner hair cell ribbon synapses
Source: Front Mol Neurosci. 2023 Sep 6;16:1248941. doi: 10.3389/fnmol.2023.1248941 (PMC10512025; doi:10.3389/fnmol.2023.1248941)
Supplement: Supplementary file 1 [file Data_Sheet_1.docx]

Supplementary Material

Synaptic activity is not required for establishing heterogeneity of inner hair cell ribbon synapses

Nare Karagulyan and Tobias Moser *

*** Correspondence:** Tobias Moser: [tmoser@gwdg.de](mailto:tmoser@gwdg.de)

# Supplementary Table

|  | **ΔF/F_o max_** | **V_h_** | **k** | **position** |
| --- | --- | --- | --- | --- |
| **ΔF/F_o max_** |  | 0.48*** | 0.17 | 0.21* |
| **V_h_** | 0.34*** |  | 0.31** | 0.23* |
| **k** | 0.09 | 0.36*** |  | -0.09 |
| **position** | 0.34*** | 0.29*** | -0.02 |  |

**Supplementary Table 1.** Correlation coefficients between synaptic properties of Vglut3^+/+^ (lower half of the matrix, shaded in grey) and Vglut3^-/-^ (upper half of the matrix, shaded in pink) IHCs. ΔF/F_o max_ shows the maximal synaptic Ca^2+^ influx, V_h_ is the voltage of half maximal activation of Ca^2+^ channels and k is the voltage sensitivity of channel activation. Position refers to the location of synapses along the pillar-modiolar axis of IHC. The correlations are consistent with the notion that modiolar synapses of Vglut3^+/+^ and Vglut3^-/-^ IHCs have stronger maximal synaptic Ca^2+^ influx (larger ΔF/F_o max_, see Fig. 2 and Fig. S1) that activates at more depolarized potentials (larger V_h_)_._ * for p < 0.05, ** p < 0.01 and *** for p < 0.001.

# Supplementary Figures


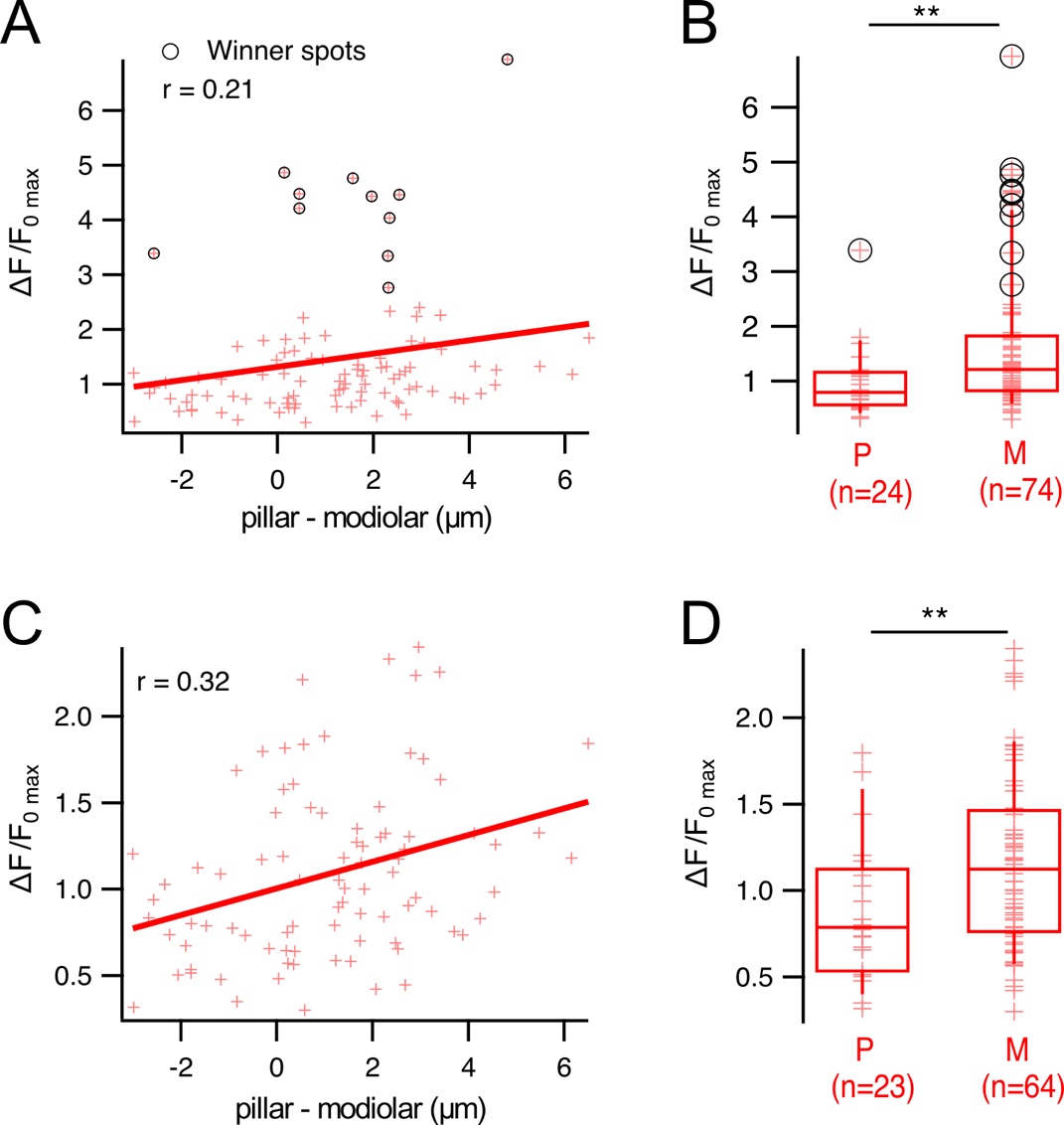


**Supplementary Figure 1 (Related to figure 2).** Contribution of “winner” spots to the spatial gradient of maximum Ca^2+^ influx in *Vglut3^-/-^* IHCs. **(A)** Single AZ maximum Ca^2+^ influx as a function of the AZ position along the modiolar-pillar axis of the cell. Black circles outline the “winner spots”. **(B)** Box plots compare ΔF/F_0max_ of pillar and modiolar AZs and show larger Ca^2+^ influx at modiolar AZs compared to the pillar AZs in *Vglut3^-/-^* IHCs (pillar: 0.98 ± 0.13, SD = 0.64, 24 spots; modiolar: 1.61 ± 0.16, SD = 1.28, 74 spots; Mann-Whitney-Wilcoxon test, p = 0.004) IHCs. Black circles outline the “winner spots”. **(C)** Spatial gradient of maximum Ca^2+^ influx without “winner” spots. **(D)** Box plots compare ΔF/F_0max_ of pillar and modiolar AZs lacking “winner” spots and show larger Ca^2+^ influx at modiolar AZs compared to the pillar AZs in *Vglut3^-/-^* IHCs (pillar: 0.88 ± 0.083, SD = 0.39, 23 spots; modiolar: 1.17 ± 0.064, SD = 0.51, 64 spots; Student’s t-test, p = 0.007).


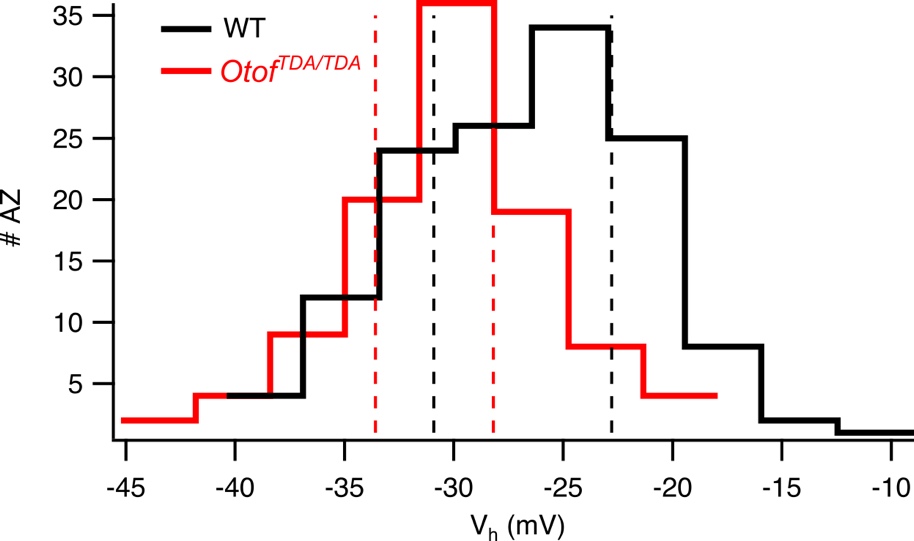


**Supplementary Figure 2 (Related to figure 6).** Distribution of voltage of half maximal activation of synaptic Ca^2+^ influx (V_h_) in WT and *Otof^TDA/TDA^* IHCs. Black and red dotted lines show the interquartile ranges of V_h_ in WT and *Otof^TDA/TDA^* IHCs respectively.
